# Mitochondrial variation in subpopulations of *Anopheles balabacensis* Baisas in Sabah, Malaysia (Diptera: Culicidae)

**DOI:** 10.1371/journal.pone.0202905

**Published:** 2018-08-23

**Authors:** Benny Obrain Manin, Chris J. Drakeley, Tock H. Chua

**Affiliations:** 1 Department of Pathobiology and Medical Diagnostics, Faculty of Medicine and Health Sciences, Universiti Malaysia Sabah, Kota Kinabalu, Sabah, Malaysia; 2 Faculty of Infectious and Tropical Diseases, London School of Hygiene and Tropical Medicine, London, United Kingdom; National Cheng Kung University, TAIWAN

## Abstract

*Anopheles balabacensis*, the primary vector of *Plasmodium knowlesi* in Sabah, Malaysia, is both zoophilic and anthropophilic, feeding on macaques as well as humans. It is the dominant *Anopheles* species found in Kudat Division where it is responsible for all the cases of *P*. *knowlesi*. However there is a paucity of basic biological and ecological information on this vector. We investigated the genetic variation of this species using the sequences of *cox*1 (1,383 bp) and *cox*2 (685 bp) to gain an insight into the population genetics and inter-population gene flow in Sabah. A total of 71 *An*. *balabacensis* were collected from seven districts constituting 14 subpopulations. A total of 17, 10 and 25 haplotypes were detected in the subpopulations respectively using the *cox*1, *cox*2 and the combined sequence. Some of the haplotypes were common among the subpopulations due to gene flow occurring between them. AMOVA showed that the genetic variation was high within subpopulations as compared to between subpopulations. Mantel test results showed that the variation between subpopulations was not due to the geographical distance between them. Furthermore, Tajima’s *D* and Fu’s *Fs* tests showed that *An*. *balabacensis* in Sabah is experiencing population expansion and growth. High gene flow between the subpopulations was indicated by the low genetic distance and high gene diversity in the *cox*1, *cox*2 and the combined sequence. However the population at Lipasu Lama appeared to be isolated possibly due to its higher altitude at 873 m above sea level.

## Introduction

*Anopheles* spp. are the only vectors of human and zoonotic malaria caused by five malaria parasite species namely *Plasmodium falciparum*, *P*. *vivax*, *P*. *malariae*, *P*. *ovale* and *P*. *knowlesi*. Approximately 70 *Anopheles* species have been known to transmit these malaria parasites in nature and 41 of them are considered as dominant vector species/species complex [1–2]. Nineteen of them are found in Asia [2] with four species viz. *Anopheles dirus*, *An*. *balabacensis*, *An*. *latens* and *An*. *introlatus* belonging to the Leucosphyrus group [3–4]. *Anopheles dirus*, a member of the Dirus complex found mainly in China, Cambodia, Vietnam, Laos and Thailand is the primary vector for human and simian malaria in Vietnam [5]. In Malaysia, *An*. *balabacensis*, *An*. *latens* and *An*. *introlatus*, all members of the Leucosphyrus complex have been incriminated as primary vectors for *P*. *knowlesi* [6–8].

*Anopheles balabacensis* is found in the forested areas of Philippines (Balabac and Palawan), Indonesia (Kalimantan, Lombok, Java and Sumba), East Malaysia (Sabah and Sarawak) and Brunei [3–4, 9]. Recent studies conducted in Sabah showed that *An*. *balabacensis* prefers to bite humans outdoors rather than indoors [10] and during the early evening with the peak biting period between 7–8 pm [8, 11–12].

Sabah has the highest incidence of *P*. *knowlesi* malaria in the world with most of the cases reported in 2013 occurring in the interior areas [13]. Records of Sabah Department of Health show that the proportion of *P*. *knowlesi* among the indigenous malaria cases for 2014–2016 was respectively 66%, 80% and 92%. Asymptomatic infection has also been detected in the community. A survey conducted in Kudat and Kota Marudu districts found that 9.8% (112/1147) of the collected blood samples were positive for *P*. *knowlesi* with the majority of the infected individuals not having a history of fever [14]. In Sabah *P*. *knowlesi* has caused most of the malaria deaths in adults [15]. Although *An*. *balabacensis* has been confirmed as the main vector for both *P*. *falciparum* [10] and *P*. *knowlesi* [8] population genetics of *An*. *balabacensis* in Sabah not been investigated.

We conducted a study on the genetic variation between subpopulations of *An*. *balabacensis* in Sabah based on the *cox*1, *cox*2 and the combined *cox*1 and *cox*2 sequences (“combined sequence”) of mitochondrial DNA. The mitochondrial DNA was used in the study as it is a suitable marker in a wide range of taxonomic, population and evolutionary studies in animals including malaria vectors [16–18]. Such population genetic analysis will help in understanding the evolution and gene flow of *An*. *balabacensis* populations.

## Materials and methods

### Collection sites

**Fig 1:**
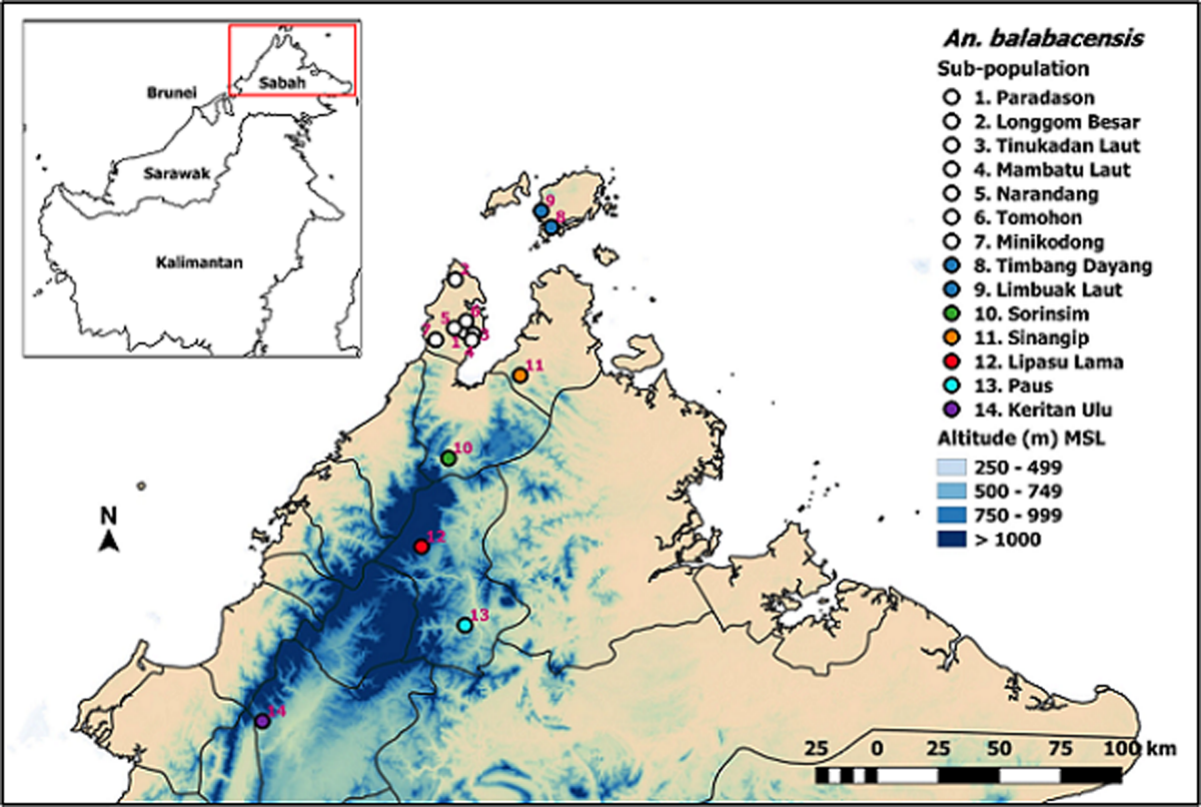
The collection sites for *An*. *balabacensis* used in this study. There were 14 sampling sites, each denoted by a different number. Seven sites were located in Kudat district, two in Banggi Island, and one each at the other five study sites. The outline of the map and the elevation map were downloaded from open source websites: http://gadm.org/country and http://www.diva-gis.org/gdata respectively, the final map was created using QGIS software version 2.18.13.

All the study sites selected had previous records of *P*. *knowlesi* cases. The inter-site distance varied from 2.4 km to 237.2 km with the GPS coordinates varying from 5.33192N - 7.21578N to 116.04140E - 117.10292E (Fig 1). The greatest inter-site distance of 237.2 km was between Limbuak Laut in Banggi Island and Keritan Ulu in mountainous Keningau district, while the shortest distance of 2.4 km was between Tinukadan Laut and Membatu Laut, both in Kudat district. The subpopulations at Limbuak Laut and Timbang Dayang are located in Banggi Island while the rest are in the main Borneo Island. The subpopulations located at the northern part of Sabah (e.g. Sorinsim, Lipasu Lama and Paus) however are separated from the Keritan Ulu subpopulation by Crocker Range and Mount Trus Madi.

### Mosquito collection and morphological identification

*Anopheles* specimens were collected from February, 2014 to September, 2016 using human landing catch method (HLC) (S1 Table). Each mosquito was kept separately inside a tube with collection details on the locality and the time caught. Any specimen still alive the next day would be killed by keeping it in the freezer (-20°C) for 3–5 minutes. The specimens were identified to species level using *Anopheles* identification keys [3,19–20] and *An*. *balabacensis* were isolated and kept individually each in a 1.5 ml microfuge tube at -30°C until use.

### PCR amplification and sequencing of the *cox*1 and *cox*2 mitochondrial gene fragments

Genomic DNA was extracted from each *An*. *balabacensis* using the DTAB-CTAB method [21] and stored at -30°C until use. Nested PCR was performed to amplify the *cox*1 and *cox*2 genes. Details of the PCR primers used are shown in S2 Table and the binding sites of the PCR primers illustrated in S1 Fig. The PCR mixture was prepared from PCR kit (Promega, USA) by mixing 10.0 μl of 5X PCR buffer, 1.0 μl of dNTPs (10 mM), 5.0 μl of MgCl_2_ (25 mM), 2.0 μl of the forward and reverse primers (10 μM), 1.0 μl of Taq DNA polymerase (5.0 U/μl), 3.0 μl of DNA template and 26.0 μl sterile dH_2_O. After the first PCR reaction was completed, 3.0 μl of the PCR product was used as a DNA template in the second PCR. The PCR reaction was performed using a thermal cycler (T100 Thermal Cycler, BioRad) with an initial denaturation at 95°C for 5 min followed by 30 cycles of denaturation at 94°C for 1 min, annealing at 55°C for 1 min, extension at 72°C for 1 min and one final extension step at 72°C for 10 min.

After the PCR was completed, the PCR products were purified using MEGA quick-spin PCR & Agarose Gel DNA Extraction System (iNtRON Biotechnology, Korea) according to the manufacturer’s procedure. The purified PCR products were analyzed on 1.5% agarose gel electrophoresis stained with RedSafe nucleic acid staining solution (iNtRON Biotechnology) and visualized using UV transilluminator. The purified PCR products were sent to AITBIOTECH (Singapore) for sequencing using forward and reverse primers (*cox*1—COIF+UAE10; *cox*2—X2F+COIIR). In order to determine the consistency of the Taq DNA polymerase, eleven PCR products of *An*. *balabacensis cox*1 and *cox*2 genes from Paradason were cloned into pGEM-TEasy vectors (Promega, USA) and the plasmids were extracted from the transformed *E*. *coli* (JM109) using DNA-spin Plasmid DNA Purification Kit (iNtRON Biotechnology, Korea) all according to the manufacturer’s procedure. The extracted plasmid was restricted using *Eco*RI restriction enzyme (Promega, USA) and the two plasmids from each gene containing the correct size of PCR amplicon were sent to AITBIOTECH, Singapore for sequencing at both directions using forward and reverse M13 primers.

### Data analysis

*Cox*1 and *cox*2 genes of 71 *An*. *balabacensis* individuals were sequenced. These sequences have been uploaded in the National Center for Biotechnology Information (NCBI) database with accession number starting from MH032606 to MH032747 (S3 Table). Subsequent analyses were performed separately using *cox*1, *cox*2 and the combined sequence.

The sequences were multi-aligned using ClustalW incorporated in the MEGA4.1 software [22]. The nucleotide sequences selected for alignment were respectively nt1509—nt2891 (1,383 bp) for *cox*1 and nt3029—nt3713 (685 bp) for *cox*2 with reference to the nucleotide sequence of *An*. *cracens* (JX219733) [23]. *Cox*1, *cox*2 and the combined sequence were translated into proteins based on the genetic code for mitochondrial DNA of *Drosophila* using DnaSP software (ver. 5.10.01) [24]. The population structure of *An*. *balabacensis* subpopulations was explored with molecular variance analysis (AMOVA) using Arlequin 3.11 [25]. The population pairwise F_ST_ values for genetic distance between the subpopulations were tested for significance as well as used for estimating gene flow, using 1,000 permutations [26]. The number of haplotypes in the subpopulations, the average number of nucleotide differences, haplotype diversity [27] and nucleotide diversity [28] were also estimated using Arlequin 3.11.

Neutrality test using Tajima’s *D* [29] and Fu’s *Fs* [30] was carried out with 1,000 simulations to analyse the randomness of the DNA sequence evolution. We further investigated the demographic expansion with mismatch analysis test using the sum of squared deviation values (SSD) and raggedness index (Rag). Estimation of the time interval for the population expansion was done using the expression, t=τ/2uk [31], where τ is the estimated number of generations since the expansion, u the mutation rate per site per generation, and k the sequence length. A mutation rate of 1.15 x 10^−8^ [32] was used.

Mantel test for isolation by distance (IBD) was performed online (http://ibdws.sdsu.edu/) with 10,000 permutations to assess the significance of correlation between genetic distance and linear geographical distance [33]. The test was conducted first, for all the subpopulations and subsequently, for the subpopulations on the main island only. The haplotype network was estimated and drawn using statistical parsimony method [34] incorporated in PopART software (http://popart.otago.ac.nz).

### Ethical clearance

This study was approved by the National Medical Research Register of the Malaysian Ministry of Health (NMRR, Ref.NMRR-12-786-13048). Consent to carry out mosquito collection was obtained from the village council or the village headman and the land owners. All volunteers who carried out mosquito collections signed informed consent forms and were provided with antimalarial prophylaxis during the study period.

## Results

### *Cox*1 and *cox*2 sequences of *An*. *balabacensis*

The *cox*1 sequence had 31.1% A, 38.4% T, 15.8% C and 14.7% G with an A + T bias of 69.5%, and can be translated into 461 amino acids. There were more mutations by transition (93.65%) than transversion (6.35%). In the transition mutations-, inter-changes between the two-ring purines: A → G (22.17%); G → A (47.03%) were more frequent than one-ring pyrimidines: C → T (17.31%); T → C (7.14%).

The *cox*2 sequence had 35.9% A, 38.4% T, 13.4% C and 12.3% G with an A + T bias of 74.3% and can be translated into 228 amino acids. Mutation by transition (86.89%) was more common than by transversion (13.11%). However in the transition, only inter-changes between two-ring purines: A → G (22.11%); G → A (64.78%) were detected.

### Mitochondrial diversity

Based on *cox*1, the subpopulations have the following genetic statistics: number of haplotypes 1–5, haplotype diversity 0–1, nucleotide diversity 0–0.00231, average number of nucleotide differences 0–3.2 and number of segregating sites 0–8 (Table 1). The subpopulation of Mambatu Laut had the highest haplotype diversity while Tomohon had the highest nucleotide diversity.

**Table 1:** MtDNA haplotypes and nucleotide diversity of *An*. *balabacensis* subpopulations based on *cox*1 sequences.

Subpopulation	No. of haplotype	No. of segregating sites	Average no. of nucleotide differences	Haplotype diversity	Nucleotide diversity
Paradason	5	5	1.055 ± 0.755	0.618 ± 0.164	0.00076 ± 0.00062
Longgom Besar	2	1	0.667 ± 0.627	0.667 ± 0.204	0.00048 ± 0.00054
Tinukadan Laut	4	3	1.400 ± 1.019	0.900 ± 0.161	0.00101 ± 0.00086
Mambatu Laut	5	5	2.200 ± 1.450	1.000 ± 0.127	0.00159 ± 0.00123
Narandang	3	5	2.667 ± 1.779	0.833 ± 0.222	0.00193 ± 0.00154
Tomohan	3	8	3.200 ± 1.979	0.700 ± 0.218	0.00231 ± 0.00167
Minikodong	2	2	1.333 ± 1.098	0.667 ± 0.314	0.00096 ± 0.00099
Timbang Dayang	5	6	3.107 ± 1.800	0.893 ± 0.086	0.00225 ± 0.00148
Limbuak Laut	4	5	1.536 ± 1.024	0.750 ± 0.139	0.00111 ± 0.00084
Sorinsim	1	0	0.000 ± 0.000	0.000 ± 0.000	0.00000 ± 0.00000
Sinangip	3	3	1.500 ± 1.121	0.833 ± 0.222	0.00109 ± 0.00097
Lipasu Lama	2	2	1.333 ± 1.098	0.667 ± 0.314	0.00096 ± 0.00099
Paus	3	2	1.167 ± 0.928	0.833 ± 0.222	0.00084 ± 0.00080
Keritan Ulu	2	1	0.667 ± 0.627	0.667 ± 0.204	0.00048 ± 0.00054
Overall	17	23	1.853 ± 1.075	0.798 ± 0.036	0.00134 ± 0.00086

Based on *cox*2, the subpopulations have the following genetic statistics: number of haplotypes 1–3, haplotype diversity 0–0.833, nucleotide diversity 0–0.00195, average number of nucleotide differences 0–1.333 and number of segregating sites 0–2 (Table 2). Sinangip subpopulation had the highest haplotype diversity while Lipasu Lama had the highest nucleotide diversity.

**Table 2:** MtDNA haplotypes and nucleotide diversity of *An*. *balabacensis* subpopulations based on *cox*2 sequences.

Subpopulation	No. of haplotype	No. of segregating sites	Average no. of nucleotide differences	Haplotype diversity	Nucleotide diversity
Paradason	2	2	0.367 ± 0.378	0.182 ± 0.144	0.00053 ± 0.00062
Longgom Besar	2	1	0.500 ± 0.519	0.500 ± 0.265	0.00073 ± 0.00091
Tinukadan Laut	1	0	0.000 ± 0.000	0.000 ± 0.000	0.00000 ± 0.00000
Mambatu Laut	3	2	0.800 ± 0.682	0.700 ± 0.218	0.00117 ± 0.00116
Narandang	2	1	0.500 ± 0.519	0.500 ± 0.265	0.00073 ± 0.00091
Tomohan	3	2	0.800 ± 0.682	0.700 ± 0.218	0.00117 ± 0.00116
Minikodong	2	1	0.667 ± 0.667	0.667 ± 0.314	0.00097 ± 0.00121
Timbang Dayang	3	2	0.679 ± 0.574	0.607 ± 0.164	0.00099 ± 0.00096
Limbuak Laut	2	1	0.571 ± 0.513	0.571 ± 0.095	0.00083 ± 0.00085
Sorinsim	1	0	0.000 ± 0.000	0.000 ± 0.000	0.00000 ± 0.00000
Sinangip	3	2	1.000 ± 0.830	0.833 ± 0.222	0.00146 ± 0.00145
Lipasu Lama	2	2	1.333 ± 1.098	0.667 ± 0.314	0.00195 ± 0.00200
Paus	2	1	0.500 ± 0.519	0.500 ± 0.265	0.00073 ± 0.00091
Keritan Ulu	2	1	0.500 ± 0.519	0.500 ± 0.265	0.00073 ± 0.00091
Overall	10	11	0.651 ± 0.511	0.495 ± 0.071	0.00095 ± 0.00083

For the combined sequence, the genetic statistics were as follows: number of haplotypes 1–5, haplotype diversity 0–1, nucleotide diversity 0 to 0.00193, average number of nucleotide differences 0–4 and number of segregating sites 0–10 (Table 3). The subpopulations of Mambatu Laut and Sinangip had the highest haplotype diversity while Tomohon had the highest nucleotide diversity.

**Table 3:** MtDNA haplotypes and nucleotide diversity of *An*. *balabacensis* subpopulations based on the combined sequence.

Subpopulation	No. of haplotype	No. of segregating sites	Average no. of nucleotide differences	Haplotype diversity	Nucleotide diversity
Paradason	5	7	1.418 ± 0.936	0.618 ± 0.164	0.00069 ± 0.00051
Longgom Besar	3	2	1.167 ± 0.928	0.833 ± 0.222	0.00056 ± 0.00054
Tinukadan Laut	4	3	1.400 ± 1.019	0.900 ± 0.161	0.00068 ± 0.00058
Mambatu Laut	5	7	3.000 ± 1.874	1.000 ± 0.127	0.00145 ± 0.00106
Narandang	3	6	3.167 ± 2.057	0.833 ± 0.222	0.00153 ± 0.00119
Tomohan	4	10	4.000 ± 2.399	0.900 ± 0.161	0.00193 ± 0.00136
Minikodong	2	3	2.000 ± 1.512	0.667 ± 0.314	0.00097 ± 0.00091
Timbang Dayang	5	8	3.786 ± 2.130	0.893 ± 0.086	0.00183 ± 0.00117
Limbuak Laut	4	6	2.107 ± 1.309	0.750 ± 0.139	0.00102 ± 0.00072
Sorinsim	1	0	0.000 ± 0.000	0.000 ± 0.000	0.00000 ± 0.00000
Sinangip	4	5	2.500 ± 1.686	1.000 ± 0.177	0.00121 ± 0.00097
Lipasu Lama	2	4	2.667 ± 1.919	0.667 ± 0.314	0.00129 ± 0.00116
Paus	3	3	1.667 ± 1.216	0.833 ± 0.222	0.00081 ± 0.00070
Keritan Ulu	3	2	1.167 ± 0.928	0.833 ± 0.222	0.00056 ± 0.00054
Overall	25	34	2.504 ± 1.366	0.889 ± 0.024	0.00121 ± 0.00073

### Haplotype diversity

**Fig 2:**
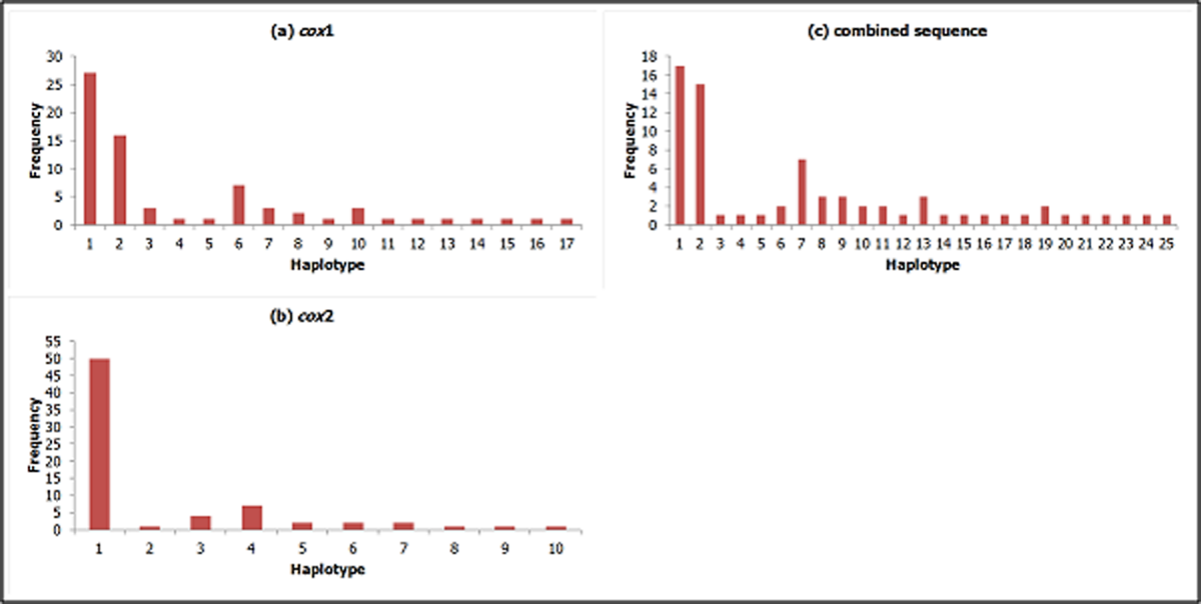
Frequency of haplotype detected in (a) *cox*1, (b) *cox*2 and (c) combined sequence across all the subpopulations of *An*. *balabacensis*.

Based on *cox*1, a total of 17 haplotypes were detected from the subpopulations (Fig 2A), with Hap_1 having the highest frequency (n = 27, 38.0%) followed by Hap_2 (n = 16, 22.5%) and Hap_6 (n = 7, 9.9%). Six haplotypes (Hap_1, Hap_2, Hap_3, Hap_6, Hap_7 and Hap_10) were shared in at least two subpopulations (S4 Table). Hap_1 was found in 12 subpopulations except in Sorinsim and Lipasu Lama, while Hap_2 was found in nine subpopulations except in Tomohon, Minikodong, Timbang Dayang, Sinangip and Lipasu Lama. Hap_6 was found in five subpopulations (Tinukadan Laut, Mambatu Laut, Minikodong, Sinangip and Lipasu Lama). Eleven haplotypes were unique, two each in Paradason, Tomohon and Timbang Dayang, but one each in Mambatu Laut, Narandang, Limbuak Laut, Lipasu Lama and Paus.

As for *cox*2, a total of ten haplotypes were observed from the subpopulations (Fig 2B). Hap_1 had the highest frequency (n = 50, 70.4%), followed by Hap_4 (n = 7, 9.9%). Five haplotypes (Hap_1, Hap_3, Hap_4, Hap_5 and Hap_6) were shared at least in two subpopulations (S4 Table). Hap_1 was found in all the subpopulations, whereas Hap_4 was found in Tomohon, Timbang Dayang, Limbuak Laut and Sinangip. Five haplotypes were unique, two of them recorded in Mambatu Laut, one each in Paradason, Lipasu Lama and Paus.

For the combined sequence, a total of 25 haplotypes were obtained (Fig 2C). Hap_1 had the highest frequency (n = 17, 23.9%), followed by Hap_2 (n = 15, 21.1%). Seven haplotypes (Hap_1, Hap_2, Hap_6, Hap_7, Hap_8, Hap_9, and Hap_13) were shared in at least two subpopulations (S4 Table). Hap_1 was found in seven subpopulations, mainly in Kudat and Keningau districts, while Hap_2 was detected in nine subpopulations except in Tomohon, Minikodong, Timbang Dayang, Sinangip and Lipasu Lama. Eighteen haplotypes were unique, three each in Paradason and Timbang Dayang, two each in Mambatu Laut, Tomohon, Lipasu Lama and Paus, and one each in Longgom Besar, Narandang, Limbuak Laut and Keritan Ulu.

### Population genetic structure

In the hierarchical AMOVA, the variance component for *cox*1 was higher within subpopulations than among subpopulations (89.84% vs 10.16%; p<0.05, Table 4). Sorinsim subpopulation had the highest F_ST_ value (0.282), while Timbang Dayang the lowest (-0.082) (S5 Table). The pairwise F_ST_ values range from -0.333 (between Longgom Besar and Keritan Ulu) to 0.600 (between Lipasu Lama and Sorinsim) with gene flow among the subpopulations varying from 0.333 to ∞ (S6 Table).

**Table 4:** AMOVA of genetic variation in *An*. *balabacensis* as detected by the *cox*1, *cox*2 and the combined sequence.

Sequence	Source of variation	Degree of freedom	Variance components	Percentage of variation	p-value
*cox*1	Among subpopulations	13	0.095	10.160	0.013
	Within subpopulations	57	0.838	89.840	
	Total	70	0.933	100.000	
*cox*2	Among subpopulations	13	0.050	15.300	0.001
	Within subpopulations	57	0.279	84.700	
	Total	70	0.329	100.000	
combined sequence	Among subpopulations	13	0.058	12.940	0.000
	Within subpopulations	57	0.391	87.060	
	Total	70	0.449	100.000	

As for *cox*2, the variance component was also higher within subpopulations compared to among subpopulations (84.70% vs 15.30%; p<0.05, Table 4). Tinukadan Laut and Sorinsim subpopulations had the highest F_ST_ value (0.323) while Lipasu Lama the lowest (-0.014) (S5 Table). The pairwise F_ST_ values range from -0.333 (between Longgom Besar and Keritan Ulu) to 0.634 (between Lipasu Lama and Tinukadan Laut) with gene flow among the subpopulations varying from 0.289 to ∞ (S7 Table).

Similarly the combined sequence also had higher variance component for within subpopulations compared to among subpopulations (87.06% vs 12.94%; p<0.05, Table 4). Sorinsim subpopulation had the highest F_ST_ value (0.304), while Mambatu Laut the lowest (0.082) (S5 Table). The pairwise F_ST_ values range from -0.212 (between Narandang and Keritan Ulu) to 0.667 (between Lipasu Lama and Sorinsim and Minikodong and Sorinsim) with gene flow among the subpopulations varying from 0.25 to ∞ (S8 Table).

The neutrality test showed four subpopulations (Paradason, Tinukadan Laut, Mambatu Laut and Sinangip) had either significant Tajima’s *D* (p<0.01) or Fu’s *Fs* (p<0.001) values for either *cox* gene or for the combined sequence. Sorinsim subpopulation had zero values for Tajima’s *D* and Fu’s *Fs* for *cox*1, *cox*2 and the combined sequence (Table 5).

**Table 5:** Neutrality tests done on *An*. *balabacensi*s subpopulations.

Subpopulation	Neutrality tests					
	*cox*1		*cox*2		combined sequence	
	Tajima's *D*	Fu's *Fs*	Tajima's *D*	Fu's *Fs*	Tajima's *D*	Fu's *Fs*
Paradason	-1.465	-1.929*	-1.430*	0.507	-1.650*	-1.204
Longgom Besar	1.633	0.540	-0.612	0.172	0.592	-0.658
Tinukadan Laut	-0.175	-1.648*	0.000	0.000	-0.175	-1.648*
Mambatu Laut	-0.562	-2.862**	-0.973	-0.829	-0.747	-2.238*
Narandang	-0.213	0.556	-0.612	0.172	-0.315	0.811
Tomohan	-1.174	1.458	-0.973	-0.829	-1.193	0.134
Minikodong	0.000	1.061	0.000	0.201	0.000	1.609
Timbang Dayang	1.598	-0.211	-0.448	-0.478	1.098	0.202
Limbuak Laut	-0.923	-0.375	1.444	0.966	-0.417	0.261
Sorinsim	0.000	0.000	0.000	0.000	0.000	0.000
Sinangip	-0.755	-0.288	-0.710	-0.887	-0.797	-1.514*
Lipasu Lama	0.000	1.061	0.000	1.061	0.000	2.022
Paus	0.592	-0.658	-0.612	0.172	0.168	-0.133
Keritan Ulu	1.633	0.540	-0.612	0.172	0.592	-0.658
Overall	-1.882**	-8.823***	-1.961**	-7.249***	-2.067**	-17.148***

Values marked with asterisk indicate significance:

*p<0.05,

**p<0.01,

***p<0.001.

Mismatch analysis showed that, the overall SSD value and the Rag index were not significant for both *cox* genes (*cox*1—SSD = 0.001, p = 0.849; Rag = 0.027, p = 0.814; *cox*2—SSD = 0.000, p = 0.8700; Rag = 0.092, p = 0.5440), but the SDD value was significant for the combined sequence (SSD = 0.044, p = 0.0060; Rag = 0.025, p = 0.962) (Table 6). At the subpopulation level, all except Tinukadan Laut and Sorinsim subpopulations show non-significant SSD values and Rag index for both genes. For the combined sequence, three subpopulations (Paradason, Lipasu Lama and Paus) showed significant SSD values, while Sorinsim subpopulation had significant SSD value and Rag index.

**Table 6:** Mismatch analysis of *An*. *balabacensi*s subpopulations.

Subpopulation	Mismatch analysis					
	*cox*1		*cox*2		combined sequence	
	SSD	Rag	SSD	Rag	SSD	Rag
Paradason	0.016	0.108	0.076	0.736	0.516***	0.155
Longgom Besar	0.090	0.556	0.022	0.250	0.037	0.250
Tinukadan Laut	0.045	0.250	0.000***	0.000***	0.045	0.250
Mambatu Laut	0.025	0.140	0.065	0.350	0.015	0.080
Narandang	0.125	0.306	0.022	0.250	0.143	0.306
Tomohan	0.153	0.470	0.065	0.350	0.058	0.190
Minikodong	0.265	1.000	0.090	0.556	0.334	1.000
Timbang Dayang	0.035	0.088	0.038	0.241	0.076	0.193
Limbuak Laut	0.010	0.055	0.043	0.347	0.070	0.228
Sorinsim	0.000***	0.000***	0.000***	0.000***	0.000***	0.000***
Sinangip	0.006	0.083	0.113	0.528	0.070	0.278
Lipasu Lama	0.265	1.000	0.265	1.000	0.370*	1.000
Paus	0.037	0.250	0.022	0.250	0.380*	1.417
Keritan Ulu	0.090	0.556	0.022	0.250	0.037	0.250
Overall	0.001	0.027	0.000	0.092	0.044**	0.025

Values marked with asterisk indicate significance:

*p<0.05,

**p<0.01,

***p<0.001.

**Fig 3:**
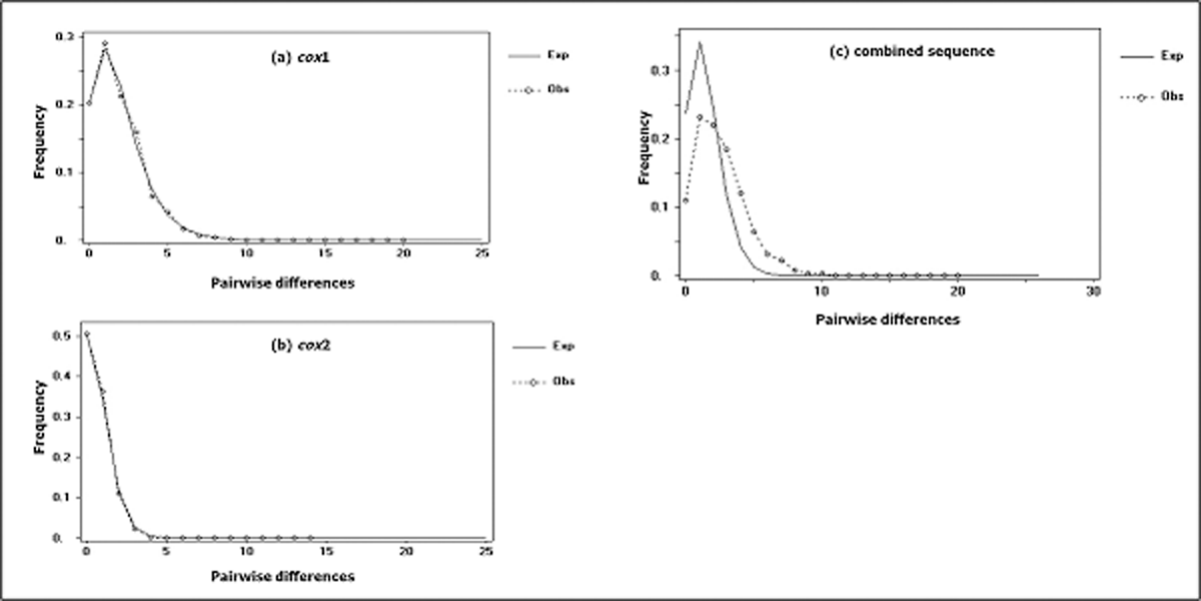
**Graphs of the mismatch distribution analysis for total populations of *An*. *balabacensis* based on (a) *cox*1, (b) *cox*2 and (c) the combined sequence.** The dotted lines represent the observed frequency of pairwise differences, and the solid lines show the expected values for the population expansion model.

The graph of the mismatch distribution for *cox*1, *cox*2 and the combined sequence showed a unimodal peak indicating the population expansion model is applicable (Fig 3). Using the expected τ values (*cox*1: 0.992; *cox*2: 0.684; combined sequence: 1.438, where τ=2ut) obtained from the expansion model, the expansion event was estimated to have taken place between 3,600 to 2,500 years ago, assuming one generation of *An*. *balabacensis* per month based on laboratory data.

**Fig 4:**
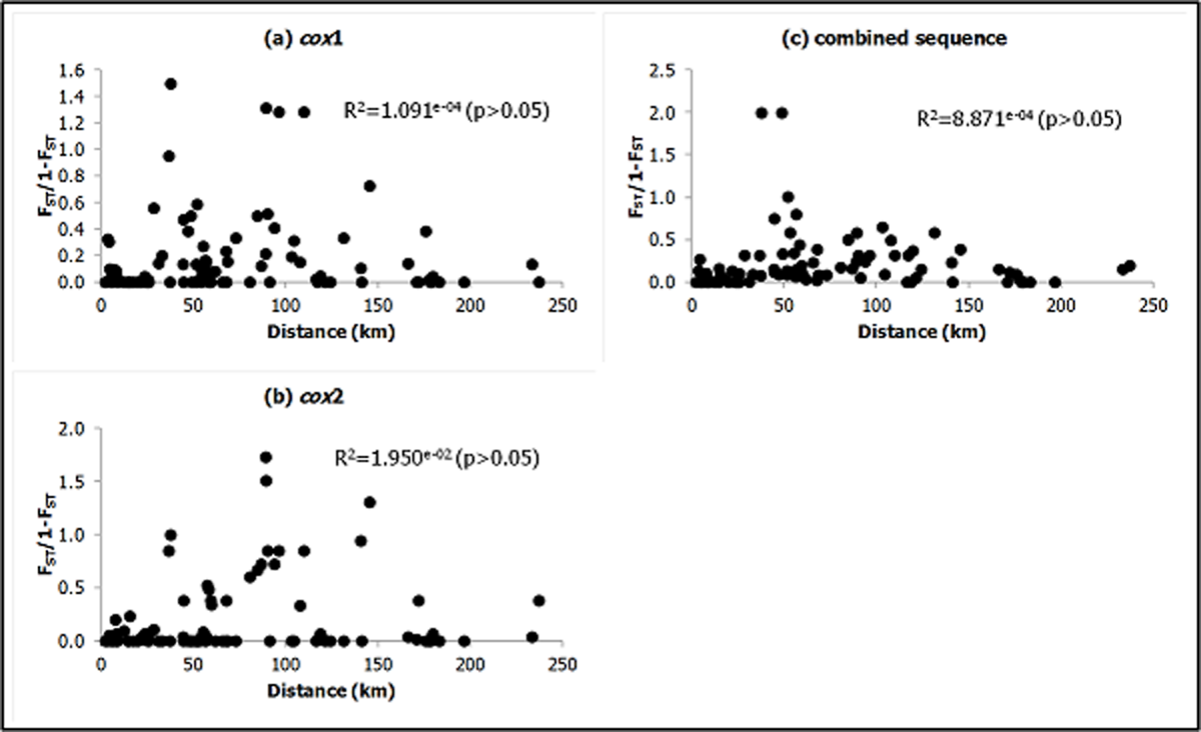
Plot of genetic distance against geographical distance between pairs of *An*. *balabacensis* subpopulations based on (a) *cox*1, (b) *cox*2 and (c) the combined sequence.

Mantel test for isolation by distance showed that the regression of the genetic distance (or linearized F_ST_ values = F_ST_/(1-F_ST_)) on geographical distance was not significant (Fig 4).

### Genealogical relationship among haplotypes

**Fig 5:**
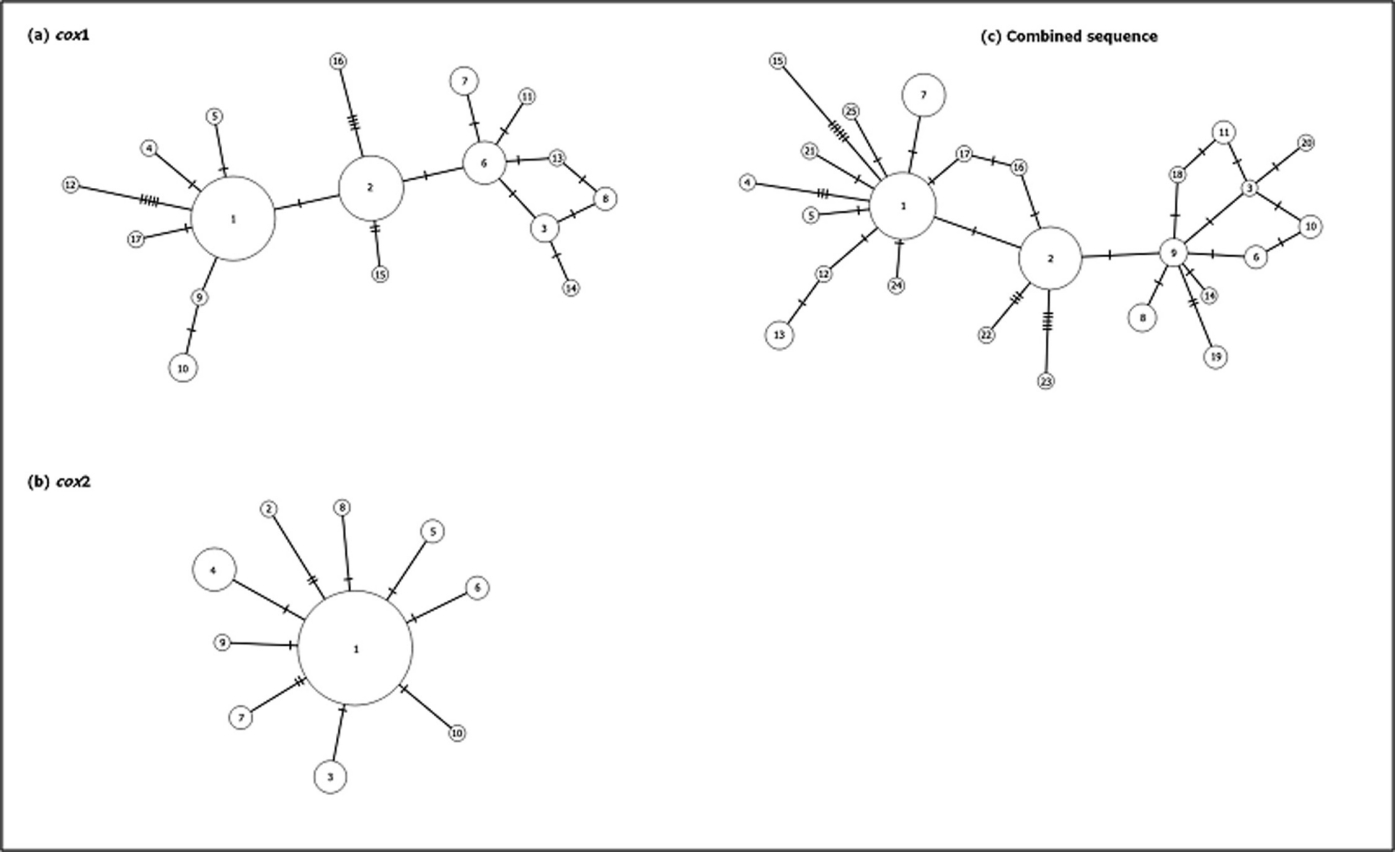
**Genealogical relationship among the haplotypes based on (a) *cox*1, (b) *cox*2 and (c) combined sequence of *An*. *balabacensis* as estimated by statistical parsimony.** Each haplotype is represented by a different number inside the circle. The size of a circle is proportional to the frequency of the haplotype. The hatch marks on the line represent mutations.

The haplotype network shows that *An*. *balabacensis* of Sabah belongs to one cluster derived from a single ancestral haplotype. Based on *cox*1 (Fig 5A), Hap_2 is considered the ancestral haplotype which is connected to the other 16 haplotypes by 1–5 mutation steps. In *cox*2 haplotype network (Fig 5B), Hap_1, the dominant haplotype is considered to be the ancestral haplotype and is connected to the other 9 haplotypes by 1–2 mutation steps. For the combined sequence, Hap_1 considered the ancestral haplotype and is connected to 24 other haplotypes by 1–6 mutation steps (Fig 5C).

**Fig 6:**
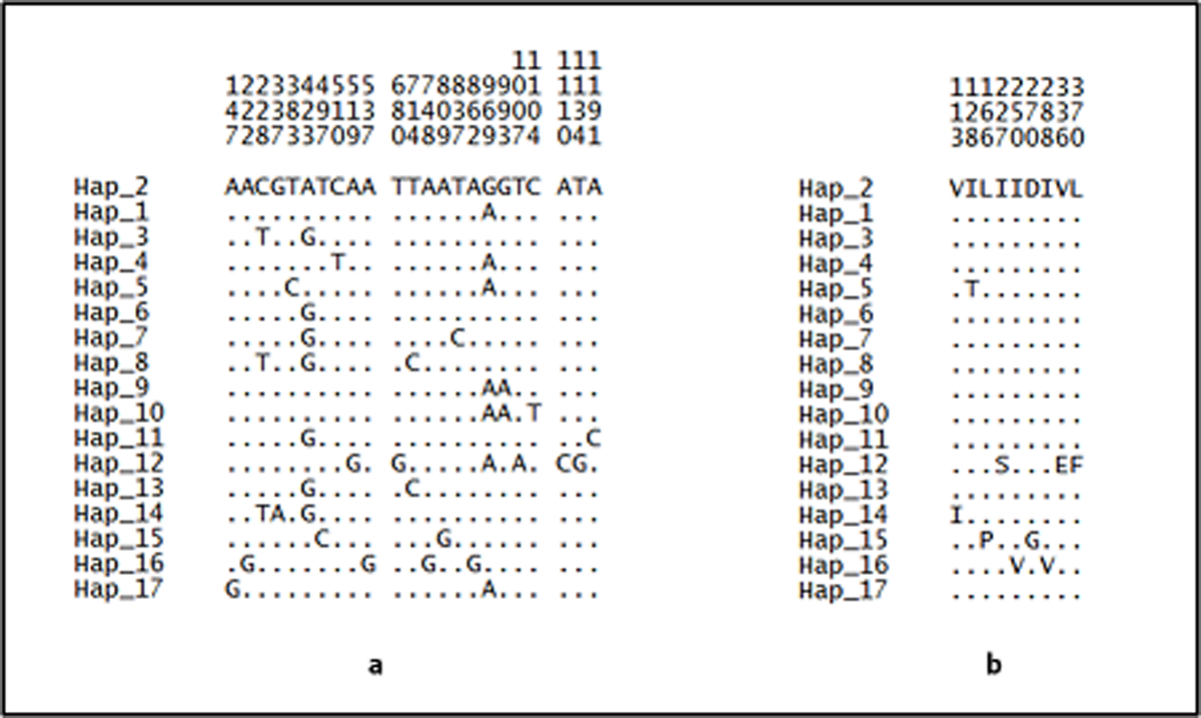
**Variable positions of (a) nucleotide and (b) amino acid in *cox*1 sequence of *An*. *balabacensis*.** The numbers shown above the sequences represent the nucleotide or amino acid position and the dots refer to the identity with reference to ancestral haplotype (Hap_2).

There are 23 mutation sites identified in *cox*1 sequence (Fig 6A), of which, 14 are synonymous while 9 are non-synonymous mutations (Fig 6B). All the synonymous mutations are located at the third codon, whereas 3 non-synonymous mutations are sited at the first codon, 5 at second codon and 1 at third codon.

**Fig 7:**
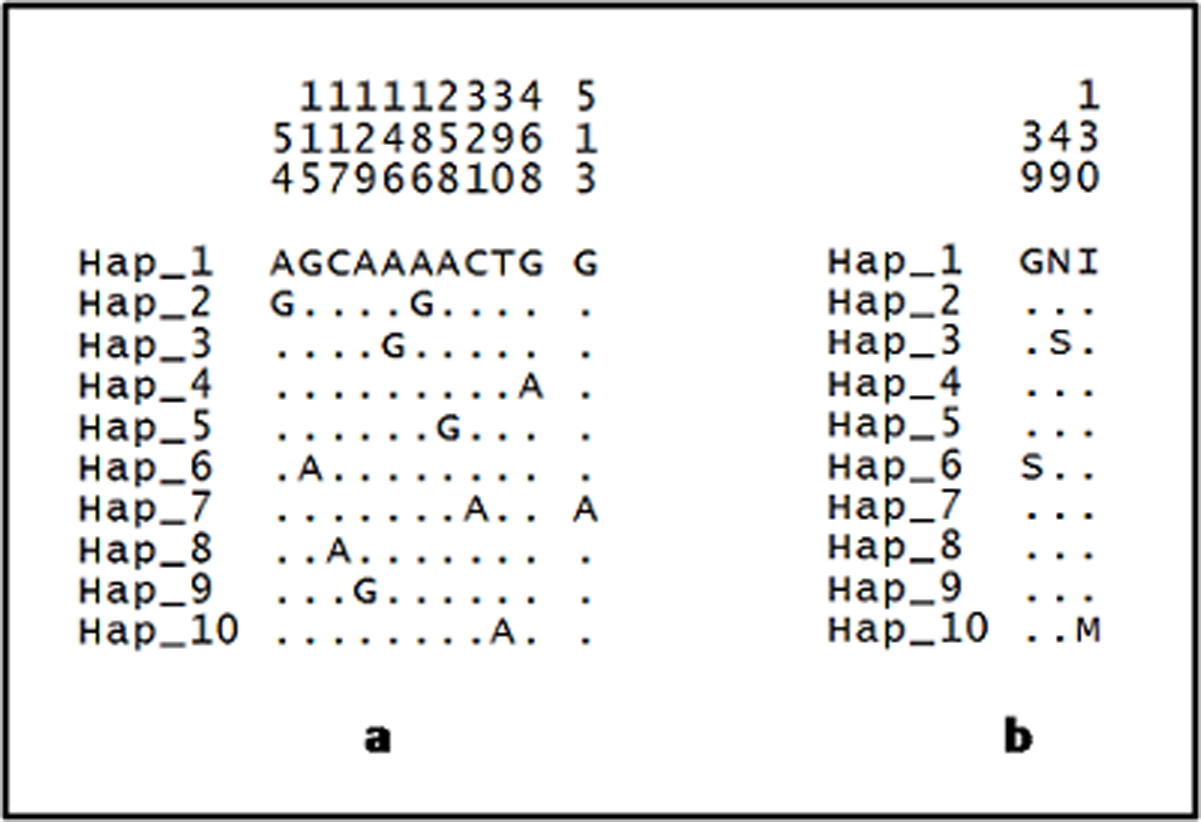
**Variable positions of (a) nucleotide and (b) amino acid in *cox*2 sequence of *An*. *balabacensis*.** The numbers shown above the sequences represent the nucleotide or amino acid position and the dots refer to the identity with reference to ancestral haplotype (Hap_1).

For *cox*2, 11 mutations are recorded in the sequence (Fig 7A), 8 of which were synonymous while 3 non-synonymous at the first, second and third codons (Fig 7B).

**Fig 8:**
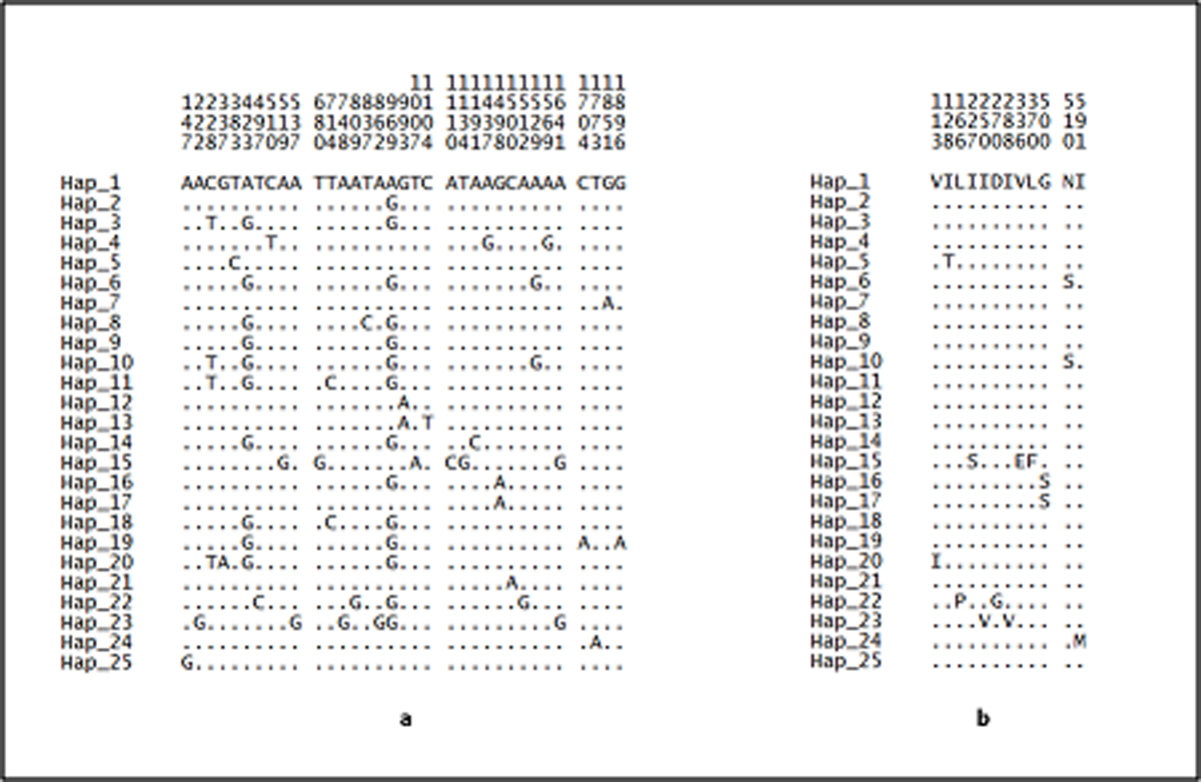
**Variable positions of (a) nucleotide and (b) amino acid in the combined sequence of *An*. *balabacensis*.** The numbers shown above the sequences represent the nucleotide or amino acid position and the dots refer to the identity with reference to ancestral haplotype (Hap_1).

As for the combined sequence, 34 mutations (22 synonymous and 12 non-synonymous) were detected (Fig 8).

## Discussion

The genetic variation of *An*. *balabacensis* populations in Sabah was explored by analyzing the partial sequence of *cox*1 (1,383 bp), *cox*2 (685 bp) and the combined sequence (2,068 bp) of the mitochondrial DNA from 71 specimens collected from 14 different sites each representing a different subpopulation.

Overall, the genetic distance between the subpopulations was low (AMOVA: F_ST_ value: *cox*1: 0.102; *cox*2: 0.153; combined sequence: 0.129), likely to be a result of inter-breeding and gene flow between the subpopulations.

Based on *cox*1 sequence the ancestral haplotype was found in five districts viz. Banggi (Limbuak Laut), Kudat (Paradason, Longgom Besar, Tinukadan Laut, Mambatu Laut, Narandang), Kota Marudu (Sorinsim), Ranau (Paus) and Keningau (Keritan Ulu) which are geographically far apart from each another, while based on the *cox*2 the ancestral was detected in all the subpopulations. However, the combined sequence showed that the ancestral was found only at two districts viz. Kudat (Paradason, Longgom Besar, Tinukadan Laut, Narandang, Tomohon and Minikodong) and Keningau (Keritan Ulu). It is unlikely that the total number of haplotypes in a subpopulation had been sampled in our study, for this would depend on the sample size [35]. However there is no obvious way to decide on required sample size based on traditional approaches [36]. The highest haplotype diversity of *cox*1 was observed in the Mambatu Laut subpopulation while the highest nucleotide diversity was in Tomohon subpopulation, both situated in the low lying areas of Kudat. Based on *cox*2 sequence, the Sinangip subpopulation has the highest haplotype diversity and Lipasu Lama has the highest nucleotide diversity, both located in higher altitude areas. The Sorinsim subpopulation had zero diversity for both sequences which could probably be due to sampling error.

Our results provide some basic information on the genetic structure of *An*. *balabacensis* especially those collected from the higher altitude areas. *Cox*1 has higher haplotype diversity and nucleotide diversity compared to *cox*2, which would suggest that *cox*1 is a more suitable molecular marker for investigating genetic variation and structure of *An*. *balabacensis*. It has been shown the number of samples and the area covered in such population study would influence the data obtained and thus the interpretation [36–37]. To obtain a better picture of the genetic variation of the population than either *cox*1 or *cox*2 alone, the complete mitochondrial DNA sequence would be required [38–39].

In general, our results show that the genetic diversity in the *cox*1, *cox*2 and the combined sequence among the *An*. *balabacensis* subpopulations was moderate to high. This indicates high gene flow between the subpopulations which may imply that *An*. *balabacensis* has high dispersal rate contributing to its success as a vector for *P*. *knowlesi*.

The sequences of both *cox*1 and *cox*2 of *An*. *balabacensis* contain a high ratio of A + T similar to other *Anopheles* species [40–42], with relatively much higher A + T at the third codon. Only mutations by transition and transversion have been detected in both sequences. The majority of these mutations are located at the third codon and higher mutation rate by transition is a common feature between the same or related species [16]. Similar finding was reported for other *Anopheles* spp.: *An*. *oswaldoi* in Brazil and for *An*. *minimus* in China and South East Asia [41–43]. However, mutations at the third codon usually do not result in altering of the amino acid composition because of the redundancy effect [43–44].

AMOVA of the sequences showed that the genetic variation in *An*. *balabacensis* subpopulations in Sabah lie within subpopulations rather than among subpopulations. Similar finding has also been recorded for other *Anopheles* species, e.g. *An*. *baimaii* (also of the Leuscosphyrus group) populations in India [45], *An*. *lesteri* [46] and *An*. *sinensis* [37,47] populations in China. In Yunnan, located in the mountainous area, the *An*. *sinensis* population was unique compared to other subpopulations in China [37]. It had been suggested that the physical barrier and the heterogeneous landscape could have inhibited gene flow between Yunnan and the other subpopulations. However for *An*. *balabacensis* in this study, the evidence is not strong enough to substantiate the conclusion that any subpopulation is isolated geographically. Nevertheless the high pairwise genetic distance and the low gene flow for the Lipasu Lama subpopulation located in the mountainous area (873 meter above the sea level), may indicate possible early stage of isolation. Another subpopulation that may also show early stage of isolation is Sorinsim, as indicated by the combined sequence analysis. It is possible that the presence of cryptic species may also contribute to the observed high pairwise of genetic distance in these two subpopulations, but this needs to be confirmed by further study.

The Limbuak Laut and Timbang Dayang subpopulations located in Banggi Island which is separated from the main island by 44 km showed small to moderate pairwise genetic distance and high gene flow, indicating that there is also sharing of genetic material between Banggi and the main island subpopulations through breeding and migration. This could possibly be achieved by *An*. *balabacensis* adults being transported unintentionally in boats or ferry along with the daily movement of people between the main island and Banggi Island.

The negative Tajima’s *D* values obtained in the overall subpopulations suggest that the DNA sequences are evolving in a non-random manner and many rare alleles are present in the subpopulations which are expanding demographically [29]. This is supported by the strong significant negative values for Fu’s *Fs* which is a more sensitive statistic for detecting deviations from neutrality and indicators for population expansion and growth [30]. A single unimodal peak (Fig 3) and the small non-significant values of the mismatch distribution analysis further support the population expansion hypothesis [48]. Furthermore, Mantel testing did not show any isolation by distance in these subpopulations indicating that the genetic variation was not caused by the geographical distance. Similar results were also observed in *An*. *dirus* and *An*. *baimaii* in South East Asia [40], *An*. *baimaii* in north-east India [45], *An*. *sinensis* [37] and *An*. *lesteri* [46] both in China, showing there is an excess of rare alleles in these populations.

The genealogy networks of *cox*1, *cox*2 and the combined sequence showed that *An*. *balabacensis* population of Sabah belongs to one cluster, suggesting that the subpopulations are expanding from a single ancestral haplotype. Based on *cox*1, this ancestral haplotype (Hap_2) was found in nine subpopulations, whereas based on *cox*2, the ancestral haplotype (Hap_1) was found in all the subpopulations. However the combined sequence showed that the ancestral haplotype (Hap_1) was found in Kudat and Keningau districts. It appears that the mutation rate of *cox*1 and *cox*2 differ [49–50], resulting in different number of haplotypes, unique haplotypes and thus different haplotype network. However, the presence of the same haplotypes in different subpopulations suggests that inter-breeding and migrations might have been occurring between the subpopulations.

This study has shown that *An*. *balabacensis* population of Sabah is undergoing population growth and expansion. The low genetic distance in the overall population of *An*. *balabacensis* based on the mitochondrial DNA indicates that there is high genetic diversity in the subpopulations, a likely consequence of inter-population migration and breeding, resulting in gene flow between the subpopulations.

## Supporting information

S1 Fig The annealing direction of the primers for *cox*1 and *cox*2 genes of *An*. *balabacensis* during the PCR amplification. (TIF)

S1 Table Details on the collection dates, sites and number of *An*. *balabacensis* collected in this study. (PDF)

S2 Table PCR primers used to amplify *cox*1 and *cox*2 genes of *An*. *balabacensis*. (PDF)

S3 Table GeneBank accession and haplotype numbers of the 71 *An*. *balabacensis* specimens. (PDF)

S4 Table Number and frequency of haplotypes observed for *cox*1, *cox*2 and the combined sequences. The haplotypes marked with asterisk (*) were detected only in one subpopulation.
(PDF)

S5 Table Fixation index (F_ST_) among populations of *An*. *balabacensis* calculated based on the *cox*1, *cox*2 and the combined sequence. (PDF)

S6 Table Pairwise genetic distance (F_ST_) and gene flow (*N*m) between subpopulations of *An*. *balabacensis* based on the *cox*1. *N*m values are shown above the diagonal while F_ST_ values below the diagonal. Values marked with asterisk indicate the genetic distances between two subpopulations are significant: *p<0.05, **p<0.01.
(PDF)

S7 Table Pairwise genetic distance (F_ST_) and gene flow (*N*m) between subpopulations of *An*. *balabacensis* based on the *cox*2. *N*m values are shown above the diagonal while F_ST_ values below the diagonal. Values marked with asterisk indicate the genetic distances between two subpopulations are significant: *p<0.05.
(PDF)

S8 Table Pairwise genetic distance (F_ST_) and gene flow (*N*m) between subpopulations of *An*. *balabacensis* based on the combined sequence. *N*m values are shown above the diagonal while F_ST_ values below the diagonal. Values marked with asterisk indicate the genetic distances between two subpopulations are significant: *p<0.05, **p<0.01, ***p<0.001.
(PDF)
