# Supplementary figures and images for: Mitochondrial variation in subpopulations of Anopheles balabacensis Baisas in Sabah, Malaysia (Diptera: Culicidae)
Source: PLoS One. 2018 Aug 23;13(8):e0202905. doi: 10.1371/journal.pone.0202905 (PMC6107281; doi:10.1371/journal.pone.0202905)

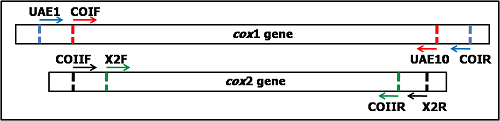

Supplement: S1 Fig — (TIF) [file pone.0202905.s001.tif]
