# Supplementary material for: Mitochondrial variation in subpopulations of Anopheles balabacensis Baisas in Sabah, Malaysia (Diptera: Culicidae)
Source: PLoS One. 2018 Aug 23;13(8):e0202905. doi: 10.1371/journal.pone.0202905 (PMC6107281; doi:10.1371/journal.pone.0202905)
Supplement: S1 Table — (PDF) [file pone.0202905.s002.pdf]

S1 Table. Details on the collection dates, sites and number of *An. balabacensis* collected in this study.

District	Site	Date	Latitude (N)	Longitude (E)	Altitude (m above sea level)	Sample size
Kudat	Paradason	Feb-Nov/2014	6.76837	116.78635	24	11
	Longgom Besar	Jul/2014	6.96205	116.75076	19	4
	Tinukadan Laut	Jun/2014	6.75930	116.81754	83	5
	Mambatu Laut	Jun/2014	6.73880	116.81166	93	5
	Narandang	May-Jun/2014	6.78297	116.74675	55	4
	Tomohan	May/2014	6.80918	116.79096	38	5
	Minikodong	Apr-May/2014	6.73986	116.67802	64	3
Banggi	Timbang Dayang	Sep-Oct/2014	7.15585	117.10292	24	8
	Limbuak Laut	Sep-Oct/2014	7.21578	117.06585	76	8
Kota Marudu	Sorinsim	Apr/2016	6.30238	116.72552	151	3
Pitas	Sinangip	Apr/2016	6.60844	116.98878	216	4
Kundasang	Lipasu Lama	May/2016	5.97634	116.62620	873	3
Ranau	Paus	Aug-Sep/2016	5.68577	116.78628	223	4
Keningau	Keritan Ulu	Aug-Sep/2016	5.33192	116.04140	481	4
