# Supplementary material for: Mitochondrial variation in subpopulations of Anopheles balabacensis Baisas in Sabah, Malaysia (Diptera: Culicidae)
Source: PLoS One. 2018 Aug 23;13(8):e0202905. doi: 10.1371/journal.pone.0202905 (PMC6107281; doi:10.1371/journal.pone.0202905)
Supplement: S2 Table — (PDF) [file pone.0202905.s003.pdf]

S2 Table. PCR primers used to amplify *cox*1 and *cox*2 genes of *An. balabacensis*.

Gene	Primer name	Sequence (5′ - 3′)	Reference
*cox*1	UAE1	GAATAATTCCCATAAATAGATTTACA	Lunt et al. 1996
	COIR	GCTTAAATTCATTGCACTAATCTGCC	This study
	COIF	TTCAGCCATTYAATCGCGACAATG	This study
	UAE10	TCCAATGCACTAATCTGCCATATTA	Lunt et al. 1996
*cox*2	COIIF	TCTAATATGGCAGATTAGTGCA	Yang et al. 2011
	X2R	TGATTTAAGAGATCATTACTTGC	Hawkes et al. 2017
	X2F	GGCAGATTAGTGCAATGAATT	Hawkes et al. 2017
	COIIR	ACTTGCTTTCAGTCATCTAATG	Yang et al. 2011

Lunt DH, Zhang DX, Szymura JM, Hewitt GM. The insect cytochrome oxidase I gene: evolutionary patterns and conserved primers for phylogenetic studies. Insect Mol Biol. 1996;5(3):153-165.

Yang M, Ma Y, Wu J. Mitochondrial genetic differentiation across populations of the malaria vector *Anopheles lesteri* from China (Diptera: Culicidae). Malar J. 2011;10:216.

Hawkes F, Manin BO, Ng SH, Torr SJ, Drakeley C, Chua TH, Ferguson HM. Evaluation of electric nets as means to sample mosquito vectors host-seeking on humans and primates. Parasites & Vectors. 2017;10:338.
