# Supplementary material for: Mitochondrial variation in subpopulations of Anopheles balabacensis Baisas in Sabah, Malaysia (Diptera: Culicidae)
Source: PLoS One. 2018 Aug 23;13(8):e0202905. doi: 10.1371/journal.pone.0202905 (PMC6107281; doi:10.1371/journal.pone.0202905)
Supplement: S3 Table — (PDF) [file pone.0202905.s004.pdf]

S3 Table. GeneBank accession and haplotype numbers of the 71 *An. balabacensis* specimens.

District	Subpopulation	Specimen code	GeneBank accession no.		Haplotype no.		
			*cox*1	*cox*2	*cox*1	*cox*2	combined sequence
Kudat	Paradason	PD615	MH032606	MH032677	Hap_1	Hap_1	Hap_1
		PD623	MH032607	MH032678	Hap_1	Hap_1	Hap_1
		PD740	MH032608	MH032679	Hap_1	Hap_1	Hap_1
		PD976	MH032609	MH032680	Hap_1	Hap_1	Hap_1
		PD991	MH032610	MH032681	Hap_2	Hap_1	Hap_2
		PD1000	MH032611	MH032682	Hap_1	Hap_1	Hap_1
		PD1015	MH032612	MH032683	Hap_1	Hap_1	Hap_1
		PD1050	MH032613	MH032684	Hap_3	Hap_1	Hap_3
		PD1217	MH032614	MH032685	Hap_1	Hap_1	Hap_1
		PD1430	MH032615	MH032686	Hap_4	Hap_2	Hap_4
		PD1538	MH032616	MH032687	Hap_5	Hap_1	Hap_5
	Longgom Besar	LBA447	MH032617	MH032688	Hap_1	Hap_6	Hap_17
		LBA450	MH032618	MH032689	Hap_2	Hap_1	Hap_2
		LBB336	MH032619	MH032690	Hap_2	Hap_1	Hap_2
		LBB340	MH032620	MH032691	Hap_1	Hap_1	Hap_1
	Tinukadan Laut	TKA274	MH032621	MH032692	Hap_7	Hap_1	Hap_8
		TKA300	MH032622	MH032693	Hap_6	Hap_1	Hap_9
		TKA327	MH032623	MH032694	Hap_1	Hap_1	Hap_1
		TKB282	MH032624	MH032695	Hap_2	Hap_1	Hap_2
		TKB286	MH032625	MH032696	Hap_2	Hap_1	Hap_2
	Mambatu Laut	MBA211	MH032626	MH032697	Hap_1	Hap_8	Hap_21
		MBA216	MH032627	MH032698	Hap_7	Hap_1	Hap_8
		MBA220	MH032628	MH032699	Hap_15	Hap_9	Hap_22
		MBA227	MH032629	MH032700	Hap_2	Hap_1	Hap_2
		MBB193	MH032630	MH032701	Hap_6	Hap_1	Hap_9

	Narandang	NRA201	MH032631	MH032702	Hap_1	Hap_1	Hap_1
		NRA209	MH032632	MH032703	Hap_1	Hap_1	Hap_1
		NRB136	MH032633	MH032704	Hap_16	Hap_5	Hap_23
		NRB140	MH032634	MH032705	Hap_2	Hap_1	Hap_2
	Tomohon	THA125	MH032635	MH032706	Hap_1	Hap_1	Hap_1
		THA132	MH032636	MH032707	Hap_1	Hap_4	Hap_7
		THA140	MH032637	MH032708	Hap_11	Hap_1	Hap_14
		THA146	MH032638	MH032709	Hap_1	Hap_1	Hap_1
		THB124	MH032639	MH032710	Hap_12	Hap_5	Hap_15
	Minikodong	MKA106	MH032640	MH032711	Hap_1	Hap_1	Hap_1
		MKA108	MH032641	MH032712	Hap_6	Hap_3	Hap_6
		MKA109	MH032642	MH032713	Hap_1	Hap_1	Hap_1
Banggi	Timbang Dayang	TD08	MH032643	MH032714	Hap_3	Hap_3	Hap_10
		TD09	MH032644	MH032715	Hap_8	Hap_1	Hap_11
		TD31	MH032645	MH032716	Hap_9	Hap_1	Hap_12
		TD32	MH032646	MH032717	Hap_8	Hap_1	Hap_11
		TD66	MH032647	MH032718	Hap_1	Hap_4	Hap_7
		TD67	MH032648	MH032719	Hap_10	Hap_1	Hap_13
		TD104	MH032649	MH032720	Hap_3	Hap_3	Hap_10
		TD105	MH032650	MH032721	Hap_10	Hap_1	Hap_13
	Limbuak Laut	LL11	MH032651	MH032722	Hap_10	Hap_1	Hap_13
		LL12	MH032652	MH032723	Hap_1	Hap_4	Hap_7
		LL28	MH032653	MH032724	Hap_2	Hap_1	Hap_2
		LL29	MH032654	MH032725	Hap_1	Hap_4	Hap_7
		LL40	MH032655	MH032726	Hap_13	Hap_1	Hap_18
		LL41	MH032656	MH032727	Hap_2	Hap_1	Hap_2
		LL63	MH032657	MH032728	Hap_1	Hap_4	Hap_7
		LL64	MH032658	MH032729	Hap_1	Hap_4	Hap_7

Kota Marudu	Sorinsim	PTX368.1	MH032659	MH032730	Hap_2	Hap_1	Hap_2
		SNX84.2	MH032660	MH032731	Hap_2	Hap_1	Hap_2
		SNX122.1	MH032661	MH032732	Hap_2	Hap_1	Hap_2
Pitas	Sinangip	SPX156.1	MH032662	MH032733	Hap_6	Hap_3	Hap_6
		SPX156.2	MH032663	MH032734	Hap_1	Hap_4	Hap_7
		SPX184.1	MH032664	MH032735	Hap_7	Hap_1	Hap_8
		SPX206.3	MH032665	MH032736	Hap_6	Hap_1	Hap_9
Kundasang	Lipasu Lama	LPX481.5	MH032666	MH032737	Hap_6	Hap_7	Hap_19
		LPX483.3	MH032667	MH032738	Hap_6	Hap_7	Hap_19
		LPX484.1	MH032668	MH032739	Hap_14	Hap_1	Hap_20
Ranau	Paus	PARFF589.1	MH032669	MH032740	Hap_1	Hap_10	Hap_24
		PARFF654.1	MH032670	MH032741	Hap_17	Hap_1	Hap_25
		PARPL591.1	MH032671	MH032742	Hap_2	Hap_1	Hap_2
		PARPL637.1	MH032672	MH032743	Hap_2	Hap_1	Hap_2
Keningau	Keritan Ulu	KRKFF663.2	MH032673	MH032744	Hap_1	Hap_1	Hap_1
		KRKPL578.1	MH032674	MH032745	Hap_1	Hap_1	Hap_1
		KRKPL581.1	MH032675	MH032746	Hap_2	Hap_1	Hap_2
		KRKPL638.1	MH032676	MH032747	Hap_2	Hap_6	Hap_16
