# Supplementary material for: Mitochondrial variation in subpopulations of Anopheles balabacensis Baisas in Sabah, Malaysia (Diptera: Culicidae)
Source: PLoS One. 2018 Aug 23;13(8):e0202905. doi: 10.1371/journal.pone.0202905 (PMC6107281; doi:10.1371/journal.pone.0202905)
Supplement: S4 Table — The haplotypes marked with asterisk (*) were detected only in one subpopulation. (PDF) [file pone.0202905.s005.pdf]

S4 Table. Number and frequency of haplotypes observed for *cox*1, *cox*2 and the combined sequences. The haplotypes marked with asterisk (*) were detected only in one subpopulation.

Subpopulation	Sample size	*cox*1		*cox*2		combined sequence	
		No. of haplotype	Haplotypes (frequency)	No. of haplotype	Haplotypes (frequency)	No. of haplotype	Haplotypes (frequency)
Paradason	11	5	1(7), 2(1), 3(1), 4(1)*, 5(1)*	2	1(10), 2(1)*	5	1(7), 2(1), 3(1)*, 4(1)*, 5(1)*
Longgom Besar	4	2	1(2), 2(2)	2	1(3), 6(1)	3	1(1), 2(2), 17(1)*
Tinukadan Laut	5	4	1(1), 2(2), 6(1), 7(1)	1	1(5)	4	1(1), 2(2), 8(1), 9(1)
Mambatu Laut	5	5	1(1), 2(1), 6(1), 7(1), 15(1)*	3	1(3), 8(1)*, 9(1)*	5	2(1), 8(1), 9(1), 21(1)*, 22(1)*
Narandang	4	3	1(2), 2(1), 16(1)*	2	1(3), 5(1)	3	1(2), 2(1), 23(1)*
Tomohan	5	3	1(3), 11(1)*, 12(1)*	3	1(3), 4(1), 5(1)	4	1(2), 7(1), 14(1)*, 15(1)*
Minikodong	3	2	1(2), 6(1)	2	1(2), 3(1)	2	1(2), 6(1)
Timbang Dayang	8	5	1(1), 3(2), 8(2)*, 9(1)*, 10(2)	3	1(5), 3(2), 4(1)	5	7(1), 10(2)*, 11(2)*, 12(1)*, 13(2)
Limbuak Laut	8	4	1(4), 2(2), 10(1), 13(1)*	2	1(4), 4(4)	4	2(2), 7(4), 13(1), 18(1)*
Sorinsim	3	1	2 (3)	1	1 (3)	1	2(3)
Sinangip	4	3	1 (1), 6 (2), 7 (1)	3	1 (2), 3 (1), 4 (1)	4	6(1), 7(1), 8(1), 9(1)
Lipasu Lama	3	2	6 (2), 14 (1)*	2	1 (1), 7 (2)*	2	19(2)*, 20(1)*
Paus	4	3	1 (1), 2 (2), 17 (1)*	2	1 (3), 10 (1)*	3	2(2), 24(1)*, 25(1)*
Keritan Ulu	4	2	1 (2), 2 (2)	2	1 (3), 6 (1)	3	1(2), 2(1), 16(1)*
Overall	71	17	1(27), 2(16), 3(3), 4(1)*, 5(1)*, 6(7), 7(3), 8(2)*, 9(1)*, 10(3), 11(1)*, 12(1)*, 13(1)*, 14(1)*, 15(1)*, 16(1)*, 17(1)*	10	1(50), 2(1)*, 3(4), 4(7), 5(2), 6(2), 7(2)*, 8(1)*, 9(1)*, 10(1)*	25	1(17), 2(15), 3(1)*, 4(1)*, 5(1)*, 6(2), 7(7), 8(3), 9(3), 10(2)*, 11(2)*, 12(1)*, 13(3), 14(1)*, 15(1)*, 16(1)*, 17(1)*, 18(1)*, 19(2)*, 20(1)*, 21(1)*, 22(1)*, 23(1)*, 24(1)*, 25(1)*
