# Supplementary material for: Mitochondrial variation in subpopulations of Anopheles balabacensis Baisas in Sabah, Malaysia (Diptera: Culicidae)
Source: PLoS One. 2018 Aug 23;13(8):e0202905. doi: 10.1371/journal.pone.0202905 (PMC6107281; doi:10.1371/journal.pone.0202905)
Supplement: S5 Table — (PDF) [file pone.0202905.s006.pdf]

S5 Table. Fixation index (F_{ST}) among populations of *An. balabacensis* calculated based on the *cox*1, *cox*2 and the combined sequence.

Subpopulation	F_{ST}		
Gene	*cox*1	*cox*2	combined sequence
Paradason	0.153	0.197	0.148
Longgom Besar	0.215	0.181	0.130
Tinukadan Laut	0.132	0.323	0.104
Mambatu Laut	0.046	0.080	0.082
Narandang	0.014	0.181	0.130
Tomohan	-0.061	0.080	0.104
Minikodong	0.163	0.154	0.180
Timbang Dayang	-0.082	0.098	0.087
Limbuak Laut	0.102	0.133	0.121
Sorinsim	0.282	0.323	0.304
Sinangip	0.131	0.039	0.095
Lipasu Lama	0.163	-0.014	0.180
Paus	0.165	0.181	0.130
Keritan Ulu	0.215	0.181	0.130
$\bar{x}$	0.117	0.153	0.138
