# Supplementary material for: Mitochondrial variation in subpopulations of Anopheles balabacensis Baisas in Sabah, Malaysia (Diptera: Culicidae)
Source: PLoS One. 2018 Aug 23;13(8):e0202905. doi: 10.1371/journal.pone.0202905 (PMC6107281; doi:10.1371/journal.pone.0202905)
Supplement: S6 Table — Nm values are shown above the diagonal while FST values below the diagonal. Values marked with asterisk indicate the genetic distances between two subpopulations are significant: *p<0.05, **p<0.01. (PDF) [file pone.0202905.s007.pdf]

S6 Table. Pairwise genetic distance (F_{ST}) and gene flow (*N*m) between subpopulations of *An. balabacensis* based on the *cox*1.

Values above the diagonal are for *N*m, while values below the diagonal are for F_{ST}. Values marked with asterisk indicate the genetic distances between two subpopulations are significant: *p<0.05, **p<0.01.

Subpopulation		1	2	3	4	5	6	7	8	9	10	11	12	13	14
Paradason	1	--	∞	1.539	1.639	5.054	24.185	∞	1.852	∞	0.851	0.894	0.380	42.755	∞
Longgom Besar	2	-0.032	--	11.875	33.750	∞	∞	∞	3.707	∞	1.500	1.300	0.389	∞	∞
Tinukadan Laut	3	0.245*	0.040	--	∞	9.444	5.227	∞	4.941	7.315	∞	∞	2.337	10.833	11.875
Mambatu Laut	4	0.234*	0.015	-0.184	--	15.000	6.136	∞	4.884	6.616	∞	∞	4.108	23.654	33.750
Narandang	5	0.090	-0.111	0.050	0.032	--	∞	∞	3.249	∞	24.000	2.500	0.970	∞	∞
Tomohan	6	0.020	-0.053	0.087	0.075	-0.017	--	∞	3.693	∞	3.038	3.516	1.222	∞	∞
Minikodong	7	-0.130	-0.167	-0.083	-0.096	-0.123	-0.190	--	∞	∞	1.000	∞	1.000	∞	∞
Timbang Dayang	8	0.213*	0.119	0.092	0.093	0.133	0.119	-0.019	--	5.532	2.617	6.222	4.797	3.560	3.707
Limbuak Laut	9	-0.020	-0.145	0.064	0.070	-0.013	-0.012	-0.201	0.083	--	3.329	2.150	0.688	∞	∞
Sorinsim	10	0.370*	0.250	-0.010	-0.051	0.020	0.141	0.333	0.160	0.131	--	1.059	0.333	3.231	1.500
Sinangip	11	0.359*	0.278	-0.158	-0.131	0.167	0.125	-0.006	0.074	0.189	0.321	--	∞	1.600	1.300
Lipasu Lama	12	0.569*	0.563**	0.176	0.109	0.340*	0.290	0.333	0.094	0.421*	0.600	-0.006	--	0.525	0.389
Paus	13	0.012	-0.222	0.044	0.021	-0.095	-0.041	-0.147	0.123	-0.098	0.134	0.238	0.488*	--	∞
Keritan Ulu	14	0.032	-0.333	0.040	0.015	-0.111	-0.053	-0.167	0.119	-0.145	0.250	0.278	0.563*	-0.222	--
