# Supplementary material for: Mitochondrial variation in subpopulations of Anopheles balabacensis Baisas in Sabah, Malaysia (Diptera: Culicidae)
Source: PLoS One. 2018 Aug 23;13(8):e0202905. doi: 10.1371/journal.pone.0202905 (PMC6107281; doi:10.1371/journal.pone.0202905)
Supplement: S7 Table — Nm values are shown above the diagonal while FST values below the diagonal. Values marked with asterisk indicate the genetic distances between two subpopulations are significant: *p<0.05. (PDF) [file pone.0202905.s008.pdf]

S7 Table. Pairwise genetic distance (F_{ST}) and gene flow (*N*m) between subpopulations of *An. balabacensis* based on the *cox*2. Values above the diagonal are for *N*m, while values below the diagonal are for F_{ST}. Values marked with asterisk indicate the genetic distances between two subpopulations are significant: *p<0.05.

Subpopulation		1	2	3	4	5	6	7	8	9	10	11	12	13	14
Paradason	1	--	16.912	∞	9.574	16.912	9.574	5.204	5.958	1.030	∞	4.682	0.331	16.912	16.912
Longgom Besar	2	0.029	--	7.500	∞	∞	∞	34.000	13.333	1.313	∞	∞	0.588	∞	∞
Tinukadan Laut	3	-0.089	0.063	--	∞	7.500	∞	2.143	27.143	0.952	∞	7.500	0.289	7.500	7.500
Mambatu Laut	4	0.050	-0.013	0.000	--	∞	∞	∞	8.883	1.463	∞	77.500	0.690	∞	∞
Narandang	5	0.029	0.000	0.063	-0.013	--	∞	34.000	13.333	1.313	∞	∞	0.588	∞	∞
Tomohan	6	0.050	-0.013	0.000	0.000	-0.193	--	∞	∞	13.846	∞	∞	0.690	∞	∞
Minikodong	7	0.088	0.015	0.189	-0.014	0.015	-0.014	--	∞	1.315	∞	∞	0.750	34.000	34.000
Timbang Dayang	8	0.077	0.036	0.018	0.053	0.036	-0.013	-0.243	--	2.500	∞	∞	0.530	13.333	13.333
Limbuak Laut	9	0.327*	0.276	0.344	0.255*	0.276	0.035	0.276	0.167	--	1.500	∞	0.382	1.313	1.313
Sorinsim	10	-0.187	-0.091	0.000	-0.132	-0.091	-0.132	0.000	-0.088	0.250	--	∞	0.500	∞	∞
Sinangip	11	0.097	0.000	0.063	0.006	0.000	-0.119	-0.268	-0.201	-0.009	-0.091	--	0.829	∞	∞
Lipasu Lama	12	0.602*	0.460	0.634	0.420	0.460	0.420	0.400	0.486*	0.567*	0.500	0.376	--	0.588	0.588
Paus	13	0.029	0.000	0.063	-0.013	0.000	-0.013	0.015	0.036	0.276	-0.091	0.000	0.460	--	∞
Keritan Ulu	14	0.029	-0.333	0.063	-0.013	0.000	-0.013	0.015	0.036	0.276	-0.091	0.000	0.460	0.000	--
