# Supplementary material for: Mitochondrial variation in subpopulations of Anopheles balabacensis Baisas in Sabah, Malaysia (Diptera: Culicidae)
Source: PLoS One. 2018 Aug 23;13(8):e0202905. doi: 10.1371/journal.pone.0202905 (PMC6107281; doi:10.1371/journal.pone.0202905)
Supplement: S8 Table — Nm values are shown above the diagonal while FST values below the diagonal. Values marked with asterisk indicate the genetic distances between two subpopulations are significant: *p<0.05, **p<0.01, ***p<0.001. (PDF) [file pone.0202905.s009.pdf]

S8 Table. Pairwise genetic distance (F_{ST}) and gene flow (*N*m) between subpopulations of *An. balabacensis* based on the combined sequence. Values above the diagonal are for *N*m, while values below the diagonal are for F_{ST}. Values marked with asterisk indicate the genetic distances between two subpopulations are significant: *p<0.05, **p<0.01, ***p<0.001.

Subpopulation		1	2	3	4	5	6	7	8	9	10	11	12	13	14
Paradason	1	--	3.789	3.727	1.864	∞	123.292	∞	1.466	1.138	0.497	1.579	0.860	1.351	∞
Longgom Besar	2	0.117	--	∞	∞	∞	13.864	5.111	3.294	4.351	6.000	5.500	1.586	∞	∞
Tinukadan Laut	3	0.118	-0.158	--	∞	∞	22.500	6.307	4.311	4.958	3.750	∞	2.071	∞	∞
Mambatu Laut	4	0.212*	-0.024	-0.134	--	16.250	9.500	3.158	8.039	4.933	1.500	∞	3.158	∞	16.250
Narandang	5	-0.078	-0.111	-0.085	0.030	--	∞	∞	3.294	2.557	0.857	5.500	1.586	10.000	∞
Tomohan	6	0.004	0.035	0.022	0.050	-0.085	--	∞	5.677	4.958	0.625	165.000	2.071	3.315	∞
Minikodong	7	-0.107	0.089	0.074	0.137	-0.136	-0.090	--	2.171	1.297	0.250	6.500	1.000	1.586	∞
Timbang Dayang	8	0.254***	0.132	0.104	0.059	0.132	0.081	0.187	--	4.842	0.771	15.579	2.171	3.294	3.294
Limbuak Laut	9	0.305***	0.103	0.092	0.092	0.164	0.092	0.278	0.094	--	1.012	24.000	1.297	4.351	2.557
Sorinsim	10	0.502*	0.077	0.118	0.250	0.368	0.444*	0.667	0.393**	0.331	--	0.667	0.250	6.000	0.857
Sinangip	11	0.241	0.083	-0.053	-0.111	0.083	0.003	0.071	0.031	0.020	0.429*	--	2.889	5.500	5.500
Lipasu Lama	12	0.368*	0.240	0.195	0.137	0.240	0.195	0.333	0.187	0.278*	0.667	0.148	--	1.586	1.586
Paus	13	0.270*	-0.111	-0.085	-0.024	0.048	0.131	0.240	0.132	0.103	0.077	0.083	0.240	--	10.000
Keritan Ulu	14	-0.078	-0.111	-0.085	0.030	-0.212	-0.085	-0.136	0.132	0.164	0.368	0.083	0.240	0.048	--
